# Knock-Down of Heterogeneous Nuclear Ribonucleoprotein A1 Results in Neurite Damage, Altered Stress Granule Biology, and Cellular Toxicity in Differentiated Neuronal Cells

**DOI:** 10.1523/ENEURO.0350-21.2021

**Published:** 2021-11-10

**Authors:** Amber Anees, Hannah E. Salapa, Patricia A. Thibault, Catherine Hutchinson, S. Austin Hammond, Michael C. Levin

**Affiliations:** 1 Office of Saskatchewan Multiple Sclerosis Clinical Research Chair, CMSNRC (Cameco MS Neuroscience Research Center), College of Medicine, University of Saskatchewan, Saskatoon, SK, S7K 0M7, Canada; 2 Department of Medicine, Neurology Division, University of Saskatchewan, Saskatoon, SK, S7N 5A2, Canada; 3 Department of Health Sciences, University of Saskatchewan, Saskatoon, SK, S7N 5A2, Canada; 4 Next-Generation Sequencing Facility, University of Saskatchewan, Saskatoon, SK, S7N 5E5, Canada; 5 Department of Anatomy, Physiology and Pharmacology, College of Medicine, University of Saskatchewan, Saskatoon, SK, S7N 5A2, Canada

**Keywords:** heterogeneous nuclear ribonucleoprotein A1, Neuro-2a cell line, neurodegenerative disease, neuronal cell damage, RNA binding protein, small interfering RNA

## Abstract

Heterogeneous nuclear ribonucleoprotein A1 (hnRNP A1) is an RNA binding protein (RBP) that is localized within neurons and plays crucial roles in RNA metabolism. Its importance in neuronal functioning is underscored from the study of its pathogenic features in many neurodegenerative diseases where neuronal hnRNP A1 is mislocalized from the nucleus to the cytoplasm resulting in loss of hnRNP A1 function. Here, we model hnRNP A1 loss-of-function by siRNA-mediated knock-down in differentiated Neuro-2a cells. Through RNA sequencing (RNA-seq) followed by gene ontology (GO) analyses, we show that hnRNP A1 is involved in important biological processes, including RNA metabolism, neuronal function, neuronal morphology, neuronal viability, and stress granule (SG) formation. We further confirmed several of these roles by showing that hnRNP A1 knock-down results in a reduction of neurite outgrowth, increase in cell cytotoxicity and changes in SG formation. In summary, these findings indicate that hnRNP A1 loss-of-function contributes to neuronal dysfunction and cell death and implicates hnRNP A1 dysfunction in the pathogenesis of neurodegenerative diseases.

## Significance Statement

Heterogeneous nuclear ribonucleoprotein A1 (hnRNP A1) plays a biologically important role in controlling gene expression and maintaining proper cellular functioning in neurons. Previous research has shown that many neurodegenerative diseases exhibit pathogenic features of hnRNP A1 dysfunction, whereby it is mislocalized from its homeostatic nuclear location to the cytoplasm resulting in loss of proper functioning. Here, we model hnRNP A1 loss-of-function in differentiated neuronal cells and show that it contributes to neuronal dysfunction and cell death. These data are important because it underscores the importance of loss-of-function models and implicates hnRNP A1 dysfunction in the pathogenesis of neurodegenerative diseases.

## Introduction

The family of RNA binding proteins (RBPs), known as heterogeneous nuclear ribonucleoproteins (hnRNPs), represent one of the most complex and diverse groups of RBPs and are among one of the most widely expressed proteins in the nucleus (Bekenstein and Soreq, 2013; Jean-Philippe et al., 2013; Thibault et al., 2021). HnRNP A1 is an abundant member of the A/B subfamily of hnRNPs that constitutes nearly 60% of the total protein mass of the hnRNP particles (Bekenstein and Soreq, 2013) and plays a vital role in gene expression through controlling mRNA stability, regulation of translation, and in processing nascent transcripts through splicing (Tavanez et al., 2012; Geuens et al., 2016). Extending beyond its mRNA functions, hnRNP A1 also mediates nuclear export, processing of miRNA and telomere biogenesis (Izaurralde et al., 1997; Ding et al., 1999; Jean-Philippe et al., 2013; Clarke et al., 2021a). These functions make hnRNP A1 an important regulator in controlling normal cellular functioning.

In neurons, precise regulation of protein homeostasis is required to maintain the highly polarized state (Thelen and Kye, 2019), in which RBPs play a key role. Dysfunction of RBPs has recently been established as a contributing factor to neuronal pathology in neurodegenerative diseases, leading to wide scale disturbances in RNA processing and activity (Belzil et al., 2013; Ling et al., 2013; Libner et al., 2020). HnRNP A1 is highly expressed in neurons and dysfunctional hnRNP A1 is a hallmark of several neurologic diseases such as Alzheimer’s disease (AD), amyotrophic lateral sclerosis, frontotemporal lobar dementia (FTLD), and recently, multiple sclerosis (MS; Donev et al., 2007; Kashima et al., 2007; Levin et al., 2017; Salapa et al., 2018, 2020a,b; Clarke et al., 2021a; Low et al., 2021). Pathologic features of hnRNP A1 dysfunction include its mislocalization from its homeostatic nuclear location to the cytoplasm, where hnRNP A1 has been found to form cytoplasmic inclusion bodies resulting in cellular dysfunction (Gami-Patel et al., 2016). In severe cases, hnRNP A1 shows reduced expression in the nucleus, resulting in a widespread disruption in mRNA metabolism (Berson et al., 2012; Honda et al., 2015). This is especially evident in analyses of cortical neurons from control and MS brains, which display a continuum of hnRNP A1 nucleocytoplasmic staining, where neurons from control patient samples exhibit physiologic nuclear hnRNP A1 localization and neurons from MS samples show pathologic hnRNP A1 nucleocytoplasmic mislocalization (Salapa et al., 2018, 2020a). Loss of hnRNP A1 from the nucleus and subsequent aggregation in the cytoplasm has led to two proposed mechanisms of disease involving either RBP loss-of-function in the nucleus or gain of toxicity in the cytoplasm, the former of which, we propose to play a principal role in the pathogenesis of neurodegeneration.

Loss-of-function of RBPs has been previously explored with another member of the hnRNP family of RBPs known as TAR-DNA-binding protein-43 (TDP-43), where nuclear clearance of TDP-43 has been shown to induce DNA double stranded break repair defects in ALS (Mitra et al., 2019) and exacerbate neurodegeneration in an AD mouse model (LaClair et al., 2016; Steinacker et al., 2019; Huang et al., 2020; Masaki et al., 2020). However, there are still critical gaps in our understanding of how loss-of-function of hnRNP A1 may impair neuronal functioning and contribute to neuronal degeneration. Knock-down of RBPs using siRNA has been used to model the RBP loss-of-function that is hypothesized to be a consequence of RBP nucleocytoplasmic mislocalization.

Therefore, in this study, we used siRNA to investigate the effect of hnRNP A1 loss-of-function in neuronal cells. RNA sequencing (RNA-seq) analysis revealed over 1500 differentially expressed (DE) transcripts following hnRNP A1 knock-down. Subsequent gene ontology (GO) analysis revealed enrichment for biological processes related to axonogenesis, neuron projection development, RNA processing, neuronal cell death, and RNP complex assembly. Additional assays confirmed hnRNP A1 knock-down negatively impacted neuronal health and affected stress granule (SG) formation, an RNP complex. These findings confirm the vital role that hnRNP A1 plays in RNA metabolism and suggests that hnRNP A1 loss-of-function leads to impaired neuronal viability by induction of cell death pathways and dysregulated RNA processing.

## Materials and Methods

### siRNA oligonucleotides

The siRNA oligonucleotide duplexes were synthesized by Integrated DNA Technologies (IDT). The siRNA sequences were as follows: scrambled/negative control siRNA (siNEG), 5′-UGGUUUACAUGUCGACAAA-3′; siRNA to hnRNP A1-set 1 (siA1 #1), 5′-GUGUAAAGUUAGUCUAUUC-3′; -set 2 (siA1 #2), 5′-GUGUGAAGUUAGAAUUCCU-3′; -set3 (siA1 #3), 5′-GGUUAUAAAAUGGUUGUUG-3′; -set4 (siA1 #4), 5′-GUAUCCAUUAUCAUGUGUA-3′. Unless mentioned otherwise, siA1 #4 was used throughout hnRNP A1 knock-down experiments and was referred to as siA1.

### Cell culture and transfection

Neuro-2a cells, a mouse neuroblastoma cell line (ATCC, CCL-131), were maintained as a monolayer in complete media consisting of DMEM, supplemented with 10% fetal bovine serum (FBS), penicillin (100 units/ml) and streptomycin (100 μg/ml) at 37°C, in a humified atmosphere containing 95% air and 5% CO_2_. To differentiate Neuro-2a cells, medium was changed to DMEM with penicillin (100 units/ml) and streptomycin (100 μg/ml), containing 2% FBS and 10 μm retinoic acid (referred to as differentiation medium). Transfection of siRNA into Neuro-2a cells was performed using Lipofectamine RNAiMAX (Invitrogen) following the manufacturer’s protocol. For siRNA knock-down and all downstream experiments, Neuro-2a cells were cultured in complete media for 24 h before siRNA transfections. Medium was then changed to differentiation medium 16 h after siRNA transfection. For initial experiments performed to test the efficacy of four different hnRNP A1 siRNA oligonucleotides, Neuro-2a cells remained undifferentiated and were harvested for Western blotting 72 h after siRNA transfection.

### Western blot analyses

Neuro-2a cells were plated onto poly-D-lysine (Sigma-Aldrich) coated six-well plates and transfected as described above and harvested for Western blotting at 72 h after siRNA transfection. Neuro-2a cells were lysed in CytoBuster (Millipore) protease extraction reagent containing protease inhibitors (Roche) as per the manufacturer’s protocol. Cell lysates were separated by SDS-PAGE before being transferred to PVDF membrane. Membranes were blocked in 10% normal goat serum for 1 h at room temperature before being placed into primary antibody for overnight incubation at 4°C. The following primary antibodies were used: mouse anti-β-actin (1:1000; Cell Signaling Technology, RRID: AB_2242334) and mouse anti-hnRNP A1 (1:1000; Millipore, RRID: AB_10562650). Membranes were washed and incubated with goat anti-mouse IgG horseradish peroxidase-conjugated secondary antibody (1:3000; Bio-Rad, RRID: AB_11125547). Membranes were developed using Clarity Western ECL substrate (Bio-Rad) and visualized using the Bio-Rad ChemiDoc system. Three biological replicates of siNEG and siA1 transfected Neuro-2a cells were harvested and run for Western blotting. Blots were analyzed in ImageJ (RRID: SCR_003070) by densitometry and normalized to β-actin signal.

### Immunocytochemistry

Neuro-2a cells were plated onto poly-D-lysine coated eight-well plates. Following transfection, cells were fixed in 3.7% formaldehyde diluted in complete medium for 15 min at 37°C and permeabilized in cold acetone for 5 min at −20°C. Cells were blocked in 100% Seablock blocking solution for 1 h at room temperature followed by incubation with primary antibodies overnight at 4°C. The following primary antibodies were used: mouse anti-hnRNP A1 (1:500; Millipore, RRID: AB_10562650), rabbit anti-hnRNP A1 (1:500; Abcam, RRID: AB_2248236), mouse anti-Ras-GTPase-activating protein SH3 domain binding protein (G3BP; 1:500; Abcam), rabbit anti-β-III-tubulin (1:1000; Sigma-Aldrich), and chicken anti-β-III-tubulin (1:500; Aves Labs, RRID: AB_2313564). Cells were washed and incubated with secondary antibodies for 30 min at room temperature. The following secondary antibodies were used: donkey anti-mouse Alexa Fluor 488 (1:1000; Jackson ImmunoResearch), goat anti-rabbit Alexa Fluor 594 (1:1000; Jackson ImmunoResearch), and donkey anti-chicken Alexa Fluor 405 (1:500; Jackson ImmunoResearch). Slides were mounted with either ProLong Gold antifade reagent with DAPI (Invitrogen) or ProLong Gold antifade reagent (Invitrogen) and imaged at 20× or 40× objective, with a 1.40 numerical aperture, on an Axio Observer 7, inverted fluorescent microscope (Carl Zeiss Canada Ltd.). Images were prepared for quantification using ZEN 3.1 Blue Edition software (Carl Zeiss Canada Ltd.).

### HnRNP A1 fluorescence analyses

Immunofluorescent images of differentiated Neuro-2a cells treated with siNEG or siA1 were stained for hnRNP A1 and ImageJ was used to assess hnRNP A1 fluorescence. To assess hnRNP A1 knock-down efficiency, nuclei were traced using DAPI to generate regions of interest (ROIs) to quantify corrected total hnRNP A1 nuclear fluorescence. For SG quantification, β-III-tubulin was used to generate ROIs to identify the entire cell and quantify corrected total hnRNP A1 cellular fluorescence. ROIs from the DAPI or β-III-tubulin images were overlaid onto hnRNP A1 images and raw integrated density was recorded for each ROIs. Background readings were recorded next to each ROI for normalization. Corrected total hnRNP A1 nuclear or cellular fluorescence was calculated using the following formula:

Corrected total A1 nuclear or cellular fluorescence = Raw Integrated Density−(Area of selected cell×Meanfluorescence of background readings).

For analyses of neurite outgrowth (Fig. 4) and SG complexity (Fig. 5), cells in the siA1-treated group were assessed for >50% knock-down of hnRNP A1. Average hnRNP A1 fluorescence of siNEG treated Neuro-2a cells were used for normalization.

### RNA harvest and library preparation

Total RNA was extracted from siNEG and siA1 transfected Neuro-2a cells using the RNeasy Midi kit (catalog #75144; QIAGEN) according to manufacturer’s protocol. RNA-seq libraries were generated from 10 ng of input RNA using the Ovation SoLo RNA-Seq Systems (catalog #0407-32; Tecan Genomics) following manufacturer’s directions.

### RNA-seq

Sequencing libraries were quantified using a Qubit 4.0 fluorometer (Invitrogen, Thermo Fisher Scientific) and Qubit 1× dsDNA HS assay (Invitrogen). The library fragment length distributions were determined using a TapeStation 4150 instrument (Agilent) with D1000 ScreenTape and reagents (Agilent). The barcoded libraries were pooled equimolar and 75-bp paired-end reads generated on a NextSeq 550 instrument (Illumina).

### Differential gene expression

The reads were extracted from each run and adapter trimmed using bcl2fastq (version 2.19.0.316; Illumina) with the following settings: “–use-bases-mask Y*,I8Y*,Y* –minimum-trimmed-read-length 0 –mask-short-adapter-reads 0.” Sequencing adapters and low-quality bases were trimmed using fastp (Chen et al., 2018) with default settings except the following: “-f 5 -Y 0 -g.” The reads were aligned to the GRCm38 mouse reference genome (Schneider and Church, 2013) using STAR (version 2.5.1b; Dobin et al., 2013) with default settings and keeping only unique alignments. Duplicate reads were identified and discarded using the NuDup tool (version 2.3; Tecan Genomics) using information from the unique molecular identifiers (UMIs) extracted from the index reads and read alignments. Gene-level expression was determined using htseq-count from the HTSeq framework (version 0.11.3; Anders et al., 2015) with default settings except for: “–nonunique all.” Genes that were DE between the control and siRNA-treated samples were identified using DESeq2 (version 1.22.2; Love et al., 2014) in R (version 3.6.1; Wilson and Norden, 2015). Significance was considered at a Benjamini–Hochberg adjusted *p* value threshold of 0.05.

### GO analyses

DE genes were analyzed using Cytoscape software (v. 3.8.2; Shannon et al., 2003; RRID: SCR_003032), including the STRING protein query to obtain protein-protein interaction networks. GO was performed using STRING enrichment analysis software (Szklarczyk et al., 2021). GraphPad Prism 9 software (RRID: SCR_002789) was used for graphical representation of GO processes.

### DE gene transcript binding analyses

Known RNA binding partners of hnRNP A1 in human were identified using CLIPdb, a database with over 300 publicly available UV-crosslinking immunoprecipitation and sequencing (CLIPseq) datasets identifying interactions between RBPs and RNA targets (Yang et al., 2015). DE genes were converted to human orthologs and compared with the data acquired from CLIPdb. Data are presented as the percent of total DE genes, upregulated DE genes, or downregulated DE genes that are known or not known to bind hnRNP A1.

### Neurite outgrowth analyses

Immunofluorescent images of differentiated Neuro-2a cells treated with siNEG and siA1 stained for β-III-tubulin were used to assess neurite outgrowth. The NeuronJ plugin (Meijering et al., 2004, RRID: SCR_002074) for ImageJ was used to trace neurites in each experimental condition using the β-III-tubulin channel. This yielded total neurite length (μm) and number of branch points for individual cells. At least 30 cells were analyzed per group per experiment and three experimental replicates were performed.

### Cell viability assay

Neuro-2a cells were plated onto poly-D-lysine coated 96-well plates. The CYQUANT lactate dehydrogenase assay (LDH; Fisher Scientific) was performed according to the manufacturer’s protocol to assess overall cell viability in differentiated Neuro-2a cells treated with siNEG or siA1 for 72 h. Three experimental replicates were performed with each condition performed in triplicate. Absorbance was measured at 680 and 490 nm using a spectrophotometer microplate reader. LDH activity was calculated through the subtraction of the absorbance at 680 nm from the absorbance at 490 nm. Percent cytotoxicity was calculated using the following formula:

$$\%\text{Cytotoxicity}=[\frac{Experimental\;group\;LDH\;activity-Spontaneous\;LDH\;activity}{Maximum\;LDH\;activity-Spontanous\;LDH\;activity}]\text{×}100$$

### SG formation analyses

Neuro-2a cells were plated onto poly-D-lysine coated eight-well plates and transfected with siNEG or siA1 for 72 h and differentiated following the established protocol. Cells were then treated with 0.5 mm sodium arsenite for 30 min and fixed and stained for immunocytochemistry. Immunofluorescent images of Neuro-2a cells treated with siNEG and siA1 stained for G3BP were used to assess SG formation. ImageJ was used to quantify the number and size (micrometers) of SGs using the G3BP channel as previously published (Clarke et al., 2021b). A total of 100 cells randomly selected in the β-III-tubulin channel were quantified per group per experiment, and three experimental replicates were performed.

### Data presentation and statistical analyses

Experimental strategy illustrations were created with BioRender.com (RRID: SCR_018361). Statistical analyses were performed using GraphPad Prism 9 software. Statistical differences were analyzed by one-tailed unpaired Student’s *t* test. The correlation between corrected total hnRNP A1 nuclear fluorescence versus neurite branch number/neurite sum length and corrected total hnRNP A1 cellular fluorescence versus number of SGs were statistically evaluated using Pearson’s correlation (PC) tests. *p* <0.05 was considered statistically significant. See Table 1 for detailed description of statistical analyses.

**Table 1:** Statistical table

Figure	Data structure	Type of test	Sample size	Statistical data
Figure 1*B*	One tailed	Unpaired *t* test	siNEG: 3 experimental replicates siA1#1: 3 experimental replicates siA1#2: 3 experimental replicates siA1#3: 3 experimental replicates siA1#4: 3 experimental replicates	siNEG vs siA1#1 *p* = 0.1471, *t* = 1.206, df = 4; siNEG vs siA1#2 *p* = 0.0277, *t* = 2.678, df = 4; siNEG vs siA1#3 *p* = 0.0041, *t* = 4.871, df = 4; siNEG vs siA1#4 *p* = 0.0011, *t* = 6.929, df = 4.
Figure 1*E*	One tailed	Unpaired *t* test	siNEG: 3 experimental replicates siA1: 3 experimental replicates	*p* = 0.0005, *t* = 8.435, df = 4, 95% CI = −1.057 to −0.5336, *r*^2^ = 0.9468, difference between means ± SEM = −0.7954 ± 0.09429
Figure 1*G*	One tailed	Unpaired *t* test	siNEG: 3 experimental replicates with over 30 cells analyzed per replicate siA1: 3 experimental replicates with over 30 cells analyzed per replicate	*p* < 0.0001, *t* = 10.50, df = 193, 95% CI = −27493 to −18797, *r*^2^ = 0.3635, difference between means ± SEM = −23145 ± 2205
Figure 4*B*	One tailed	Unpaired *t* test	siNEG: 3 experimental replicates with over 90 cells analyzed in total Severe siA1: 3 experimental replicates with 20 cells analyzed in total	*p* = 0.0002, *t* = 3.644, df = 116, 95% CI = −2.890 to −0.8547, *r*^2^ = 0.1027, difference between the means ± SEM = −1.872 ± 0.5139
Figure 4*D*	One tailed	Unpaired *t* test	siNEG: 3 experimental replicates with over 90 cells analyzed in total Severe siA1: 3 experimental replicates with 20 cells analyzed in total	*p* = 0.0002, *t* = 3.671, df = 116, 95% CI = −62.74 to −18.76, *r*^2^ = 0.1041, difference between the means ± SEM = −40.75 ± 11.10
Figure 4*F*	One tailed	Unpaired *t* test	siNEG: 3 experimental replicates siA1: 3 experimental replicates	*p* = 0.0221, *t* = 2.900, df = 4, 95% CI = 0.1822–8.404, *r*^2^ = 0.6776
Figure 5*B*	One tailed	Unpaired *t* test	siNEG: 3 experimental replicates with over 290 cells analyzed in total Severe siA1: 3 experimental replicates with 141 cells analyzed in total	*p* < 0.0001, *t* = 7.480, df = 437, 95% CI = −2.641 to −1.542, *r*^2^ = 0.1135, difference between the means ± SEM = −2.091 ± 0.2796
Figure 5*C*	One tailed	PC test	siA1: analyzed 300 cells for their hnRNP A1 fluorescence and SG number; each dot represents a single cell	*p* < 0.0001, *r* = 0.2825, *r*^2^ = 0.07982, 95% CI = 0.1749–0.3835
Figure 5*D*	One tailed	Unpaired *t* test	siNEG: 3 experimental replicates with over 290 cells analyzed in total siA1: 3 experimental replicates with 141 cells analyzed in total	*p* < 0.0001, *t* = 6.494, df = 443, 95% CI = −0.5998 to −0.3211, *r*^2^ = 0.08691, difference between the means ± SEM = −0.4605 ± 0.07091

### Data accessibility

The RNA-seq reads are available under NCBI BioProject PRJNA766065.

## Results

### Knock-down of hnRNP A1 in Neuro-2a cells as a loss-of-function model

**Figure 1.**
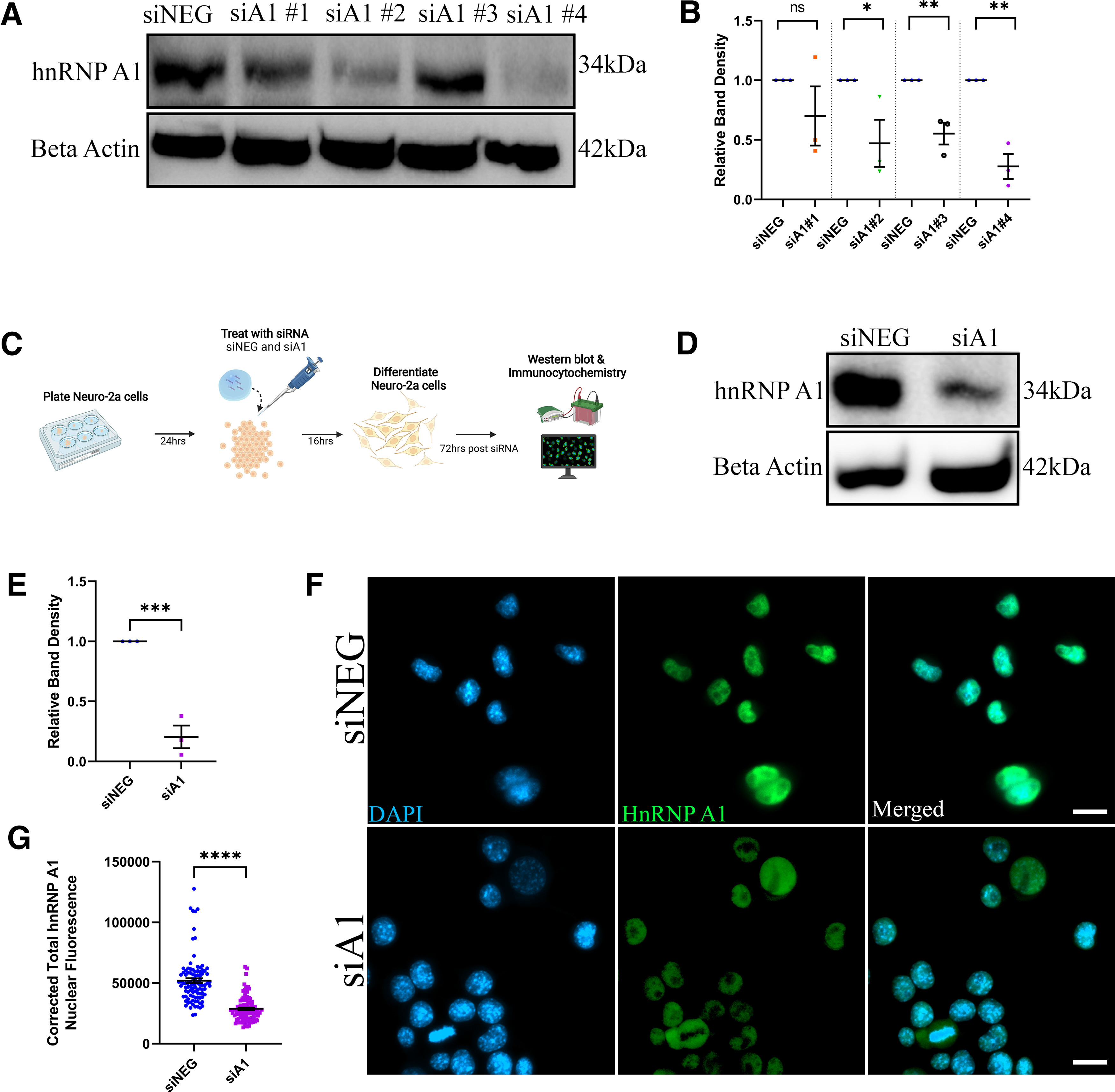
Efficient knock-down of hnRNP A1 in differentiated Neuro-2a cells. ***A***, Undifferentiated Neuro-2a cells were treated with four different siA1 duplex oligonucleotides for 72 h, which showed varying degrees of hnRNP A1 knock-down. ***B***, Quantification of ***A*** demonstrating siA1#4 was the most potent siA1 duplex oligonucleotide to significantly decrease hnRNP A1 expression compared with siNEG. Unpaired *t* test (ns = non-significant, *p < 0.05, **p < 0.01); *n* = 3 biological replicates. Data are plotted as mean ± SEM. ***C***, Neuro-2a transfection, differentiation and data collection protocol. ***D***, Protein from differentiated Neuro-2a cells treated with either siNEG or siA1 for 72 h were assayed by Western blotting for hnRNP A1 and β-actin. ***E***, Band densitometry of Western blottings as in ***D*** demonstrates a significant decrease in hnRNP A1 protein expression after 72 h of treatment with siA1 as compared with siNEG. Unpaired *t* test (***p < 0.001); *n* = 3 biological replicates. Data are plotted as mean ± SEM. ***F***, Confirmation of decreased hnRNP A1 expression (green) following treatment with siA1 using immunocytochemistry. Scale bar: 20 μm. ***G***, Corrected total hnRNP A1 nuclear fluorescence was measured using ImageJ. Cells in the siA1 condition demonstrated significant reduction in hnRNP A1 expression as compared with siNEG-treated cells. Unpaired *t* test (****p < 0.0001); *n* = 3 biological replicates. Individual cell values (*n* = 30 cells per replicate) are plotted as mean ± SEM.

To optimize siRNA knock-down of hnRNP A1, four different siRNA oligonucleotides were tested (siA1 #1–#4) for hnRNP A1 knock-down efficiency. When compared with Neuro-2a cells treated with a negative control non-targeting siRNA (siNEG), siA1 #4 was found to be the most potent siRNA to significantly reduce hnRNP A1 expression (Fig. 1*A*,*B*). Therefore, siA1#4 was chosen for subsequent experiments (herein referred to as siA1), which was used to knock down hnRNP A1 expression before Neuro-2a differentiation (Fig. 1*C*). We confirmed hnRNP A1 knock-down in differentiated Neuro-2a cells by Western blotting (Fig. 1*D*,*E*) and immunofluorescence (Fig. 1*F*,*G*) and found that differentiated Neuro-2a cells treated with siA1 showed significantly decreased hnRNP A1 expression as compared with siNEG-treated cells.

### HnRNP A1 knock-down leads to dysregulation of transcripts involved in splicing, neuronal function, cell death, and RNP complex assembly

**Figure 2.**
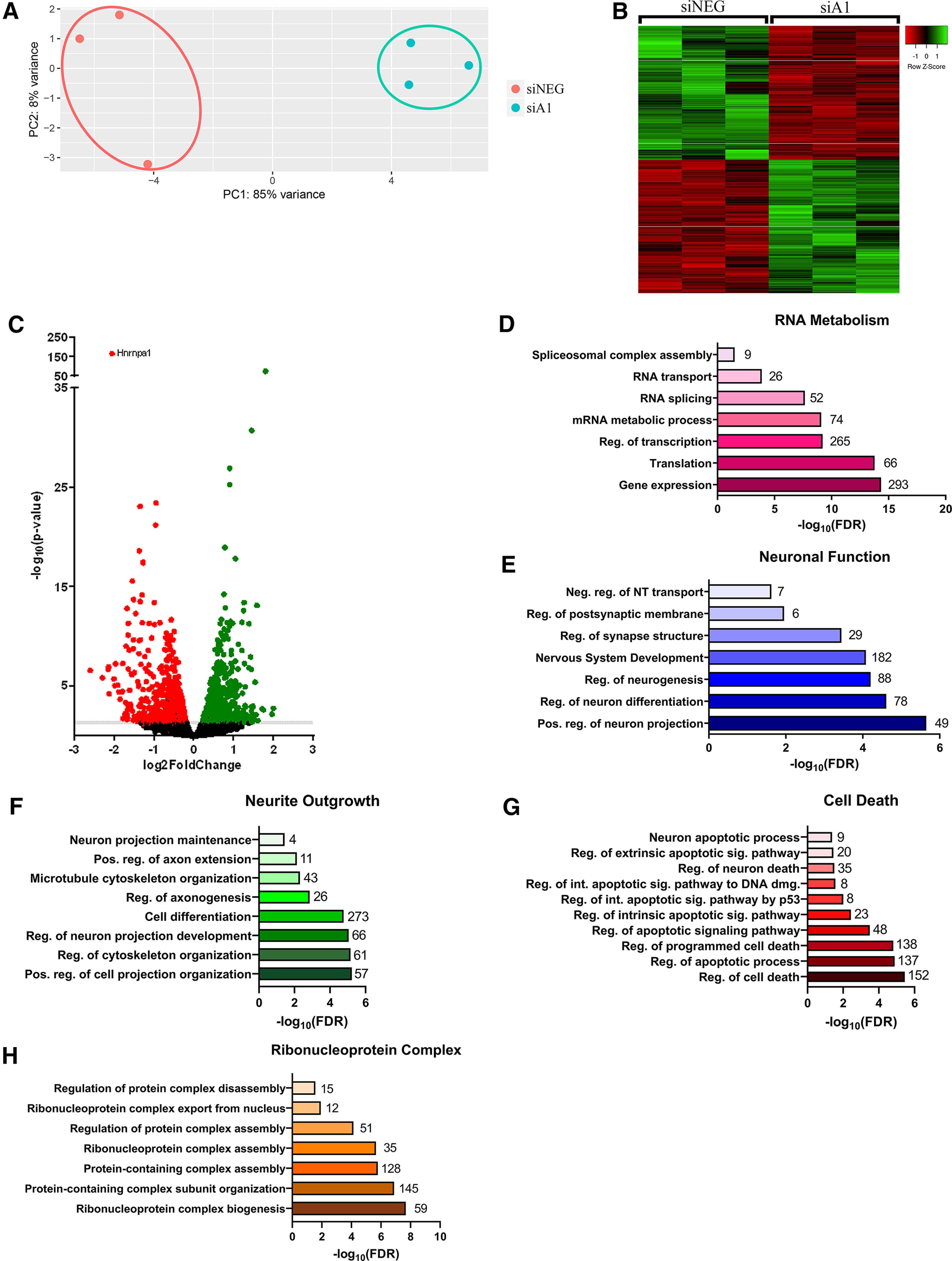
RNA-seq analysis of hnRNP A1 knock-down in differentiated Neuro-2a cells. ***A***, PCA analysis of log transformed normalized RNA-seq data showing that siA1 and siNEG formed distinct clusters with strong intercluster separation. ***B***, Heatmap of DE transcripts plotted as normalized count values for siNEG-treated (*n* = 3) and siA1-treated (*n* = 3) cells. ***C***, Volcano plot of siA1-treated samples (siA1 vs siNEG) illustrating significantly upregulated (green dots) and downregulated (red dots) transcripts. Non-DE transcripts are represented as black dots; *p* threshold of 0.05 is displayed in gray. See Extended Data Figure 2-1 for list of significant DE genes. ***D–G***, GO enrichment analysis of DE genes identified GO terms related to RNA metabolism (***D***), neuronal functions (***E***), neuronal morphology (***F***), cell death (***G***), and RNP complex (***H***). Values at the end of each bar represent number of DE genes in each GO process. Data are presented as -log_10_false discovery rate (FDR) values, which represent p-values adjusted for multiple tests by Benjamini–Hochberg procedure. See Extended Data Figure 2-2 for list of significantly enriched GO terms from biological processes.

To identify cellular processes and pathways affected by hnRNP A1 loss-of-function, we performed RNA-seq using total RNA isolated from siNEG and siA1 treated Neuro-2a cells. Principal component analysis (PCA) revealed that the siNEG and siA1 samples formed distinct clusters with strong intercluster separation (Fig. 2*A*). Subsequent analyses revealed a total of 1561 DE transcripts following hnRNP A1 knock-down (Fig. 2*B*,*C*; Extended Data Fig. 2-1). Of these transcripts, 782 were significantly downregulated, and 779 significantly upregulated (Fig. 2*B*,*C*). Furthermore, the gene with the most statistically significant difference between the siNEG and siA1 samples was hnrnpa1, which supports the efficacy of the siRNA treatment (Fig. 2*C*).

10.1523/ENEURO.0350-21.2021.f2-1 Extended Data Figure 2-1 List of DE genes with Ensembl transcript IDs, gene symbols, fold changes for each transcript, log2(fold change), *p* values, adjusted *p* value (*p*adj), -log(*p*adj), change direction, and significance. Download Figure 2-1, XLS file.

10.1523/ENEURO.0350-21.2021.f2-2 Extended Data Figure 2-2 Significantly enriched GO terms from biological processes grouped into the following categories: RNA metabolism, neuronal function, neurite outgrowth, cell death, and RNP complex. Categories include number of genes (# genes), category, description, false discovery rate (FDR) values, -log(FDR value), genes, *p* values, and term name. Download Figure 2-2, XLS file.

Next, we performed GO term analysis to identify pathways and processes that were enriched within the DE transcripts. GO analysis revealed an enrichment of transcripts whose functions were related to RNA metabolism, including regulation of transcription, RNA splicing, RNA transport, and spliceosomal complex assembly (Fig. 2*D*; Extended Data Fig. 2-2). Additionally, we found that hnRNP A1 knock-down led to an enrichment of DE transcripts related to neuronal functioning, including regulation of neurogenesis, nervous system development, and regulation of synapse structure or activity (Fig. 2*E*). We also found an enrichment of transcripts whose functions were related to neurite outgrowth and cell death (Fig. 2*F*,*G*), such as neuron projection development, regulation of axonogenesis, neuron projection maintenance, and regulation of programmed cell death. Lastly, we found an enrichment of transcripts whose functions related to RNP complex assembly, which included RNP complex biogenesis, RNP complex export from nucleus, and regulation of protein complex disassembly (Fig. 2*H*).

### HnRNP A1 binding on transcripts dysregulated by hnRNP A1 knock-down

**Figure 3.**
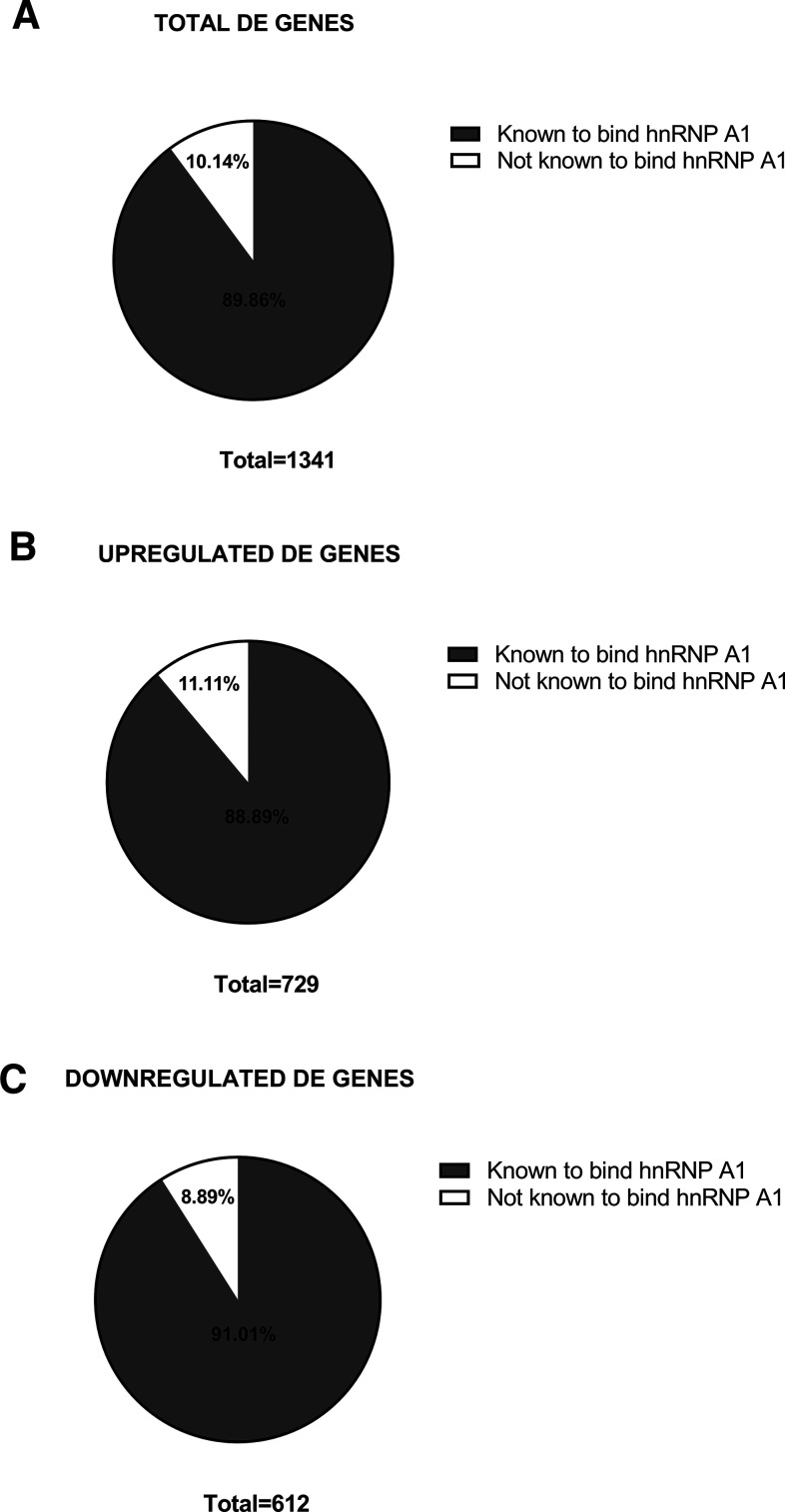
HnRNP A1 binding to DE genes. ***A***, Pie chart representing the subset of DE genes with human orthologs (*n* = 1341) that had previously been shown to be known hnRNP A1 binding targets (89.86%) and those that had not (10.14%). ***B***, Pie chart representing subset of upregulated DE genes with human orthologs (*n* = 729) that had previously been shown to be known hnRNP A1 binding targets (88.89%) and those that had not (11.11%). ***C***, Pie chart representing subset of downregulated DE genes with human orthologs (*n* = 612) that had previously been shown to be known hnRNP A1 binding targets (91.01%) and those that had not (8.89%).

Next, we investigated the relationship between our identified DE transcripts and transcripts that have been previously reported to bind hnRNP A1. To achieve this, we used CLIPdb (Yang et al., 2015) a database with over 300 publicly available UV-CLIPseq datasets identifying RBP-RNA recognition domains, structural preferences, and, of particular interest in these experiments, transcripts that have been shown to bind each RBP. As there are no recorded datasets for transcripts that have been shown to bind hnRNP A1 in mice, we converted our DE transcripts to human orthologs and overlaid our dataset with the available data from human studies identifying hnRNP A1-bound transcripts. Out of our 1561 DE mouse genes, 1341 of those genes were found to have human orthologs. Further, we found that 1205 out of 1341 DE genes (89.86%) had previously been found to bind hnRNP A1 (Fig. 3*A*) with 88.89% of the upregulated transcripts (Fig. 3*B*) compared with 91.01% of the downregulated (Fig. 3*C*) having previously been found to bind hnRNP A1. This result suggests that the majority of identified DE transcripts might be dysregulated because of the inability of hnRNP A1 to bind these transcripts and properly process them.

### Knock-down of hnRNP A1 negatively impacts neuronal phenotype

**Figure 4.**
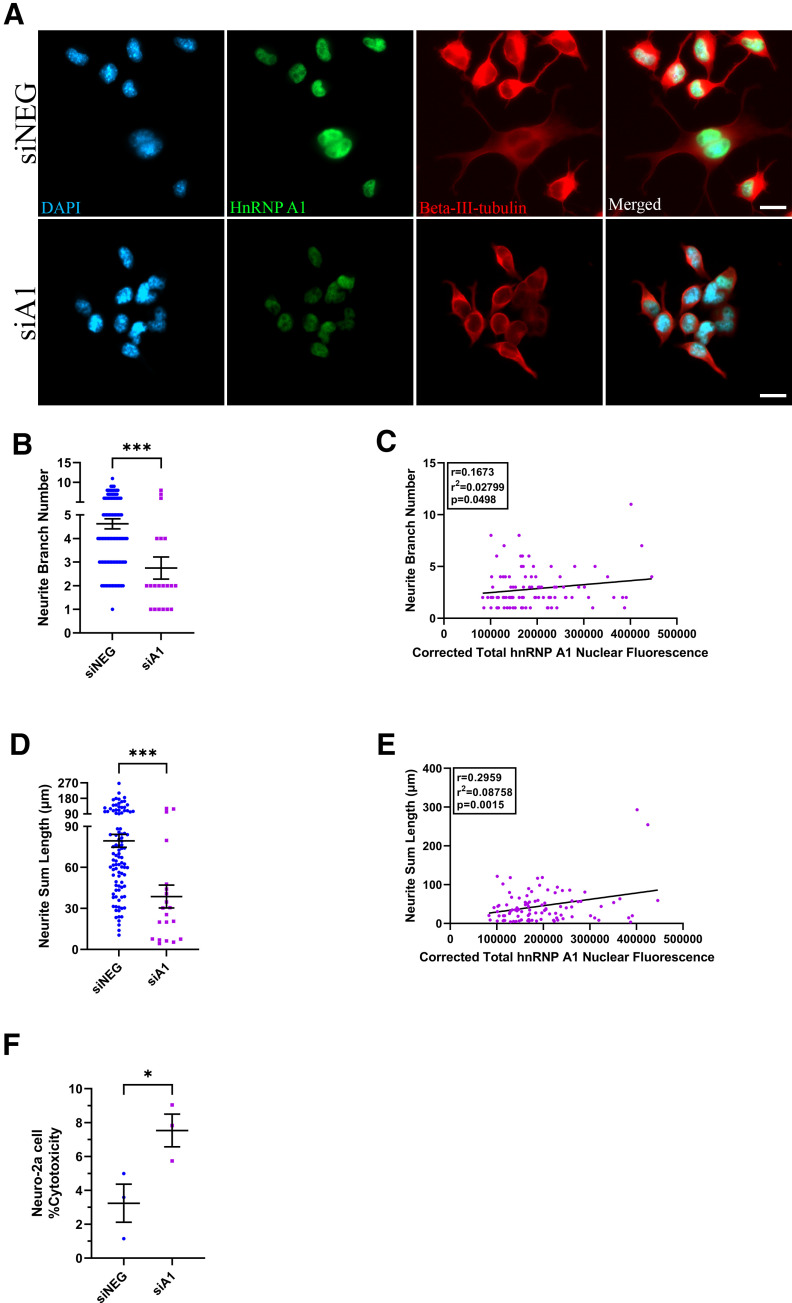
Effect of hnRNP A1 knock-down on neuronal health. ***A***, Immunofluorescent images of Neuro-2a cells stained for DAPI (blue), hnRNP A1 (green), and β-III-tubulin (red) to identify neurites. Cells in the siNEG condition have more neurites that appear longer as compared with the siA1 condition. Scale bar: 20 μm. ***B***, Neurites were traced in the β-III-tubulin channel in ImageJ using the NeuronJ plugin as described in Materials and Methods. Quantification revealed that siA1-treated Neuro-2a cells have significantly fewer neurite branches as compared with the siNEG condition. Unpaired *t* test (***p < 0.001); *n* = 3 biological replicates. Individual cell values (*n* = 30 cells per replicate for siNEG; *n* = 20 cells with >50% knock-down for siA1) are plotted as the mean ± SEM. ***C***, Corrected total hnRNP A1 nuclear fluorescence of Neuro-2a cells treated with siA1 correlates with neurite branch number. PC test (*r* = 0.167, *r*^2^ = 0.02,799, *p* = 0.0498); *n* = 3 biological replicates. Individual cell values (*n* = 30 cells per replicate) are plotted. ***D***, Neuro-2a cells treated with siA1 have significantly shorter neurites as compared with the siNEG condition. Unpaired *t* test (***p < 0.001); *n* = 3 biological replicates. Individual cell values (*n* = 30 cells per replicate for siNEG; *n* = 20 cells with >50% knock-down for siA1) are plotted as mean ± SEM. ***E***, Corrected total hnRNP A1 nuclear fluorescence of Neuro-2a cells treated with siA1 correlated with neurite sum length. PC test (*r* = 0.2959, *r*^2^ = 0.08,758, *p* = 0.0015); *n* = 3 biological replicates. Individual cell values (*n* = 30 cells per replicate) are plotted. ***F***, HnRNP A1 knock-down significantly increased cellular cytotoxicity as compared with siNEG-treated cells as measured by the CYQUANT LDH cytotoxicity assay. Unpaired *t* test (*p < 0.05); *n* = 3 biological replicates. Data are plotted as mean ± SEM.

The ability of neuronal cells to develop and project neurites from their cell bodies is a well-established measure of their health and function (Radio and Mundy, 2008; Harrill et al., 2013). Given the GO analyses that showed siA1 treated differentiated Neuro-2a cells altered transcripts related to neurite outgrowth and cell death, we posited that these cells would display evidence of altered neurite phenotype and cell death. First, we used neurite outgrowth as a measure of overall neuronal health. When compared with siNEG-treated cells, siA1-treated cells exhibited a significant reduction in neurite branching and neurite sum length (Fig. *4A*,*B*,*D*). There was a weak but statistically significant correlation between hnRNP A1 nuclear expression with neurite branching and neurite length (Fig. 4*C*,*E*). Next, we assessed the effect of hnRNP A1 knock-down on neuronal viability. Using a LDH cytotoxicity assay, we found that knock-down of hnRNP A1 caused increased cytotoxicity in siA1-treated differentiated Neuro-2a cells as compared with siNEG-treated cells (Fig. 4*F*). Taken together, these results indicate that hnRNP A1 knock-down has a detrimental effect on neuronal morphology resulting in increased cell cytotoxicity.

### Decreased hnRNP A1 expression affects SG formation

**Figure 5.**
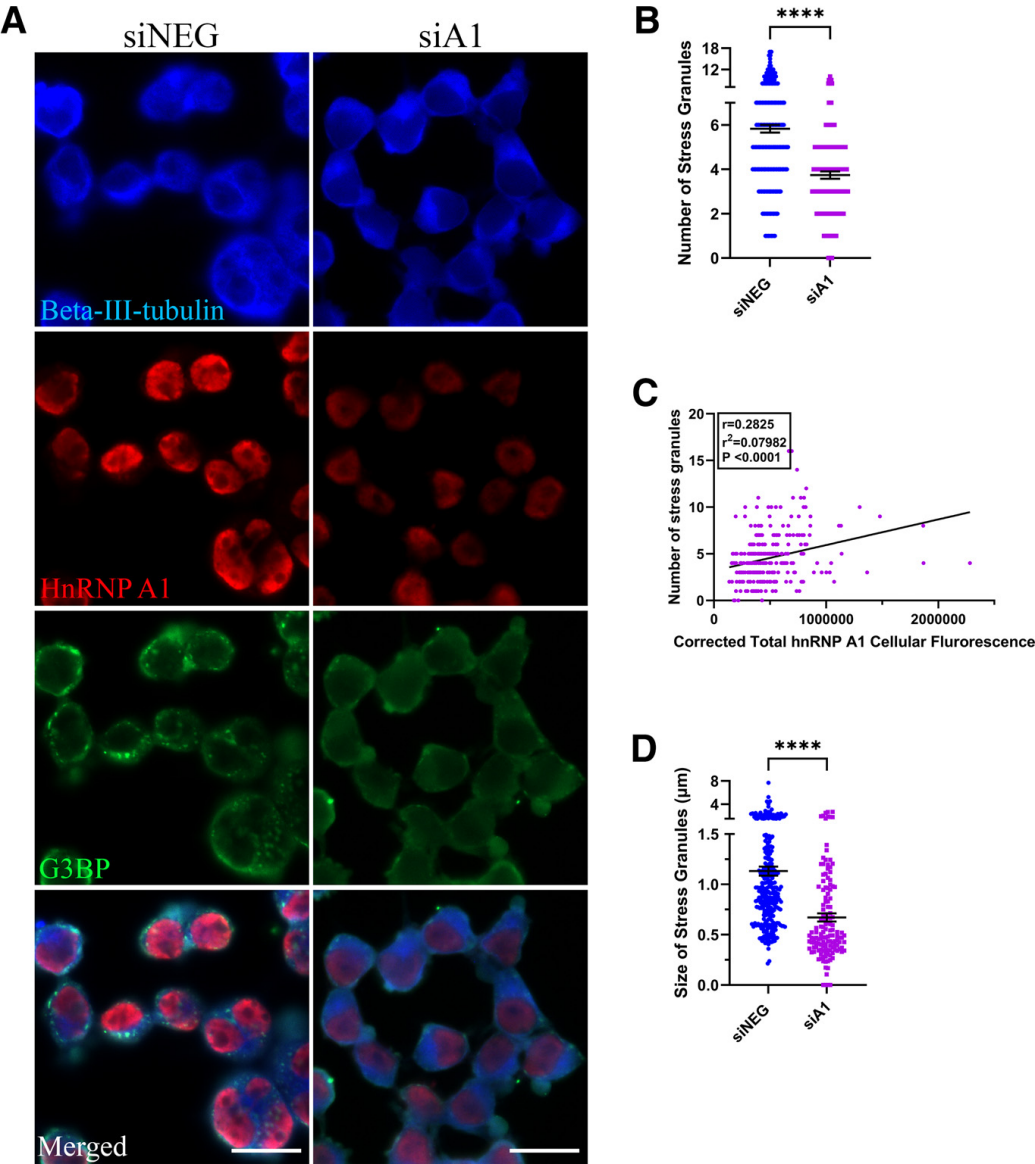
HnRNP A1 knock-down affects SG formation. ***A***, Immunofluorescent images of differentiated Neuro-2a cells treated with siNEG or siA1 for 72 h followed by 30-min treatment with sodium arsenite. Cells are stained for β-III-tubulin (blue), hnRNP A1 (red), and G3BP (green) to identify SGs. Cells in the siNEG condition have significantly more punctate-like G3BP^+^ granules as compared with the siA1 condition. Scale bar: 20 μm. ***B***, Quantification revealed that sodium arsenite-treated Neuro-2a cells in the siA1 condition form significantly fewer SGs as compared with the siNEG condition. Unpaired *t* test (****p < 0.0001); *n* = 3 biological replicates. Individual cell values (*n* = 90 cells per replicate for siNEG; *n* = 141 cells with >50% knock-down for siA1) are plotted as mean ± SEM. ***C***, HnRNP A1 cell fluorescence of Neuro-2a cells treated with siA1 followed by sodium arsenite treatment correlates with number of SGs. PC test (*r* = 0.2825, *r*^2^ = 0.07,982, *p* < 0.0001); *n* = 3 biological replicates. Individual cell values (*n* = 90 cells per replicate) are plotted. ***D***, Quantification revealed that sodium arsenite-treated Neuro-2a cells in the siA1 condition have significantly smaller SGs as compared with the siNEG condition. Unpaired *t* test (****p < 0.0001); *n* = 3 biological replicates. Individual cell values (*n* = 90 cells per replicate for siNEG; *n* = 141 cells with >50% knock-down for siA1) are plotted as mean ± SEM.

From our GO term analysis, we found that RNP complex biogenesis was a biological process that was enriched for in our DE gene dataset. SGs are a form of RNP complexes that assemble during stress and hnRNP A1 has been found in SGs under several stress conditions (Purice and Taylor, 2018; Clarke et al., 2021a,b). Therefore, we wanted to examine whether hnRNP A1 knock-down would affect SG formation and complexity, so we used a classic stressor, sodium arsenite, to promote SG formation. We found that hnRNP A1 knock-down resulted in a significant reduction in the number of SGs that formed in Neuro-2a cells (Fig. 5*A*,*B*), which correlated with hnRNP A1 expression in cells (Fig. 5*C*). Additionally, we found that hnRNP A1 knock-down significantly reduced the size of the SGs that did form, as compared with the control (Fig. 5*D*). These results underscore the biologically important role of hnRNP A1 during the stress response and the formation of SGs.

## Discussion

Mislocalization of RBPs, including hnRNP A1, have been shown to be key pathologic features of neurologic diseases with a significant neurodegenerative component, such as ALS, FTLD, and MS (Salapa et al., 2018, 2020a; Steinacker et al., 2019; Huang et al., 2020; Masaki et al., 2020). Several hypotheses explain a link between dysfunctional RBPs, and specifically their mislocalization, and neurodegeneration. Two of the most common include cytoplasmic gain of toxicity and nuclear loss-of-function (Hanson et al., 2012; Harley et al., 2021). The former suggests that there is a gain of toxicity in the cytoplasm when an RBP is mislocalized leading to consequences such as abnormal binding and processing of cytosolic RNA targets. In the latter hypothesis, it is thought that RBP loss-of-function is deleterious leading to lack of proper RNA processing, disrupted splicing regulation, and transcriptional control. Here, we investigated the latter hypothesis by modeling hnRNP A1 loss-of-function in differentiated Neuro-2a cells, a neuronal cell line, by employing siRNA knock-down, a technique commonly used to model RBP loss-of-function in cells (Iguchi et al., 2009; Fiesel et al., 2010, 2011).

After establishing a model of hnRNP A1 loss-of-function in differentiated Neuro-2a cells, we performed RNA-seq to elucidate the molecular consequences of this. We identified over 1500 DE genes, which were found to be enriched for numerous biological processes related to RNA metabolism and neuronal morphology and health. Furthermore, almost 90% of the identified DE transcripts have previously been shown to be bound by hnRNP A1, suggesting that perturbations in the identified transcripts might be because of lack of binding and proper processing by hnRNP A1.

Several of the identified biological processes related to RNA metabolism, including RNA splicing, regulation of transcription, and RNA transport are established roles of hnRNP A1. For example, hnRNP A1 has been shown to interact with and cause the degradation of inhibitory subunit of the nuclear factor ĸB (IĸBα; Sahu et al., 2014), consequently resulting in the activation of nuclear factor ĸB (NF-ĸB), a transcriptional factor involved in regulating the immune response (Hayden and Ghosh, 2008). Furthermore, the role for hnRNP A1 in alternative splicing is well-documented, including differential splicing of HIV-1 trans-activator of transcription (HIV-Tat; Zahler et al., 2004). While our data confirms a role for hnRNP A1 in these processes in neuronal cells, it also provides insight into RNA disturbances found in many neurodegenerative diseases, which might be linked to hnRNP A1 loss-of-function. Given that neurons require tight homeostatic regulation of transcripts, large scale RNA disturbances, as observed in many neurologic diseases, result in dire consequences on neuronal health. Dysregulation of RBPs such as hnRNP A1, are often considered precipitating factors in these neurodegenerative diseases (Clarke et al., 2021a) and could contribute to the wide scale disruption in RNA metabolism within neurons.

While literature suggests that hnRNP A1 is important in neuronal health, there have been no studies that directly demonstrate this relationship. In this study, GO analyses revealed several biological processes related to neuronal morphology, health, and development that were affected by hnRNP A1 knock-down, including axonogenesis, nervous system development, and regulation of neuron projection. Some of these terms establish an important role for hnRNP A1 in neuronal functioning, such as regulation of neuron projection, regulation of neurogenesis and regulation of neurotransmitter transport, while others, such as nervous system development, confirm previous findings. For example, previous literature has demonstrated embryonic lethality in hnRNP A1 knock-out mice, confirming the importance of hnRNP A1 in nervous system development (Liu et al., 2017). However, the effects of hnRNP A1 knock-down on axonogenesis and neuron projection have not been explored. Therefore, we examined neurite outgrowth and branching following hnRNP A1 knock-down and found decreased neurite outgrowth and branching in siA1-treated cells as compared with controls. Additionally, neurite outgrowth and branching were found to positively correlate with hnRNP A1 nuclear expression in cells. We confirmed the detrimental effect of hnRNP A1 knock-down on neuronal cells by performing a LDH cytotoxicity assay, which showed increased cytotoxicity in siA1 treated differentiated Neuro-2a cells. This demonstrates that hnRNP A1 plays an active role in neurite outgrowth; however, the exact mechanism by which this occurs remains to be elucidated. One possibility is that hnRNP A1 regulates transcripts important for these processes. For example, TDP-43 has been shown to be involved in the regulation of Rho family GTPases (Iguchi et al., 2009) and histone deacetylases (Fiesel et al., 2011), key components for neurite outgrowth in neuronal cells. A similar mechanism may also be employed by hnRNP A1. Furthermore, hnRNP A1 has been shown to interact with TDP-43 (Deshaies et al., 2018) and interestingly, Tardbp, the transcript for TDP-43, was significantly downregulated following hnRNP A1 knock-down.

Finally, GO analysis revealed enrichment for the biological process RNP complex assembly. Intriguingly, SGs, a RNP complex, are a feature of dysfunctional RBPs in disease and are often found to be co-localized with mislocalized RBPs in the cytoplasm of cells (Vanderweyde et al., 2013). SGs contain a diverse mix of translationally stalled RNAs and RBPs during times of stress and can easily disassemble following the removal of the stressor (Vanderweyde et al., 2013; Protter and Parker, 2016). HnRNP A1 has been shown to associate with SGs under stress conditions and co-localizes with SGs under pathologic conditions (Guil et al., 2006; Vanderweyde et al., 2013; Salapa et al., 2018). Therefore, we investigated the effect of hnRNP A1 knock-down on SG formation. We found a significant reduction in both SG number and size following hnRNP A1 knock-down, suggesting that hnRNP A1 either directly or indirectly significantly influences SG kinetics. These findings align with previous studies showing that hnRNP A1 associates with SGs in HeLa cells and that knock-down of hnRNP A1 impacts the cell’s ability to recover from stress (Guil et al., 2006).

Several studies have shown the propensity for cytoplasmic hnRNP A1 to form insoluble aggregates, affect SG dynamics, and abnormally bind RNA targets (Kim et al., 2013; Bolognesi et al., 2016; Salapa et al., 2018). These observations suggest that hnRNP A1 contributes to neurodegeneration through cytoplasmic gain of toxicity mechanisms. However, to our knowledge, none of these studies have provided a mechanistic link between cytoplasmic hnRNP A1 and neurodegeneration. In contrast, our findings offer mechanistic insight into how hnRNP A1 loss-of-function contributes to neurodegeneration. However, it is most likely a combination of both cytoplasmic gain of toxicity and nuclear loss-of-function of hnRNP A1 that results in neurodegeneration in neurologic diseases.

In summary, we demonstrate that neuronal knock-down of hnRNP A1, used here to model hnRNP A1 loss-of-function, leads to the dysregulation of transcripts related to RNA metabolism, neuronal morphology, and cell death. We further confirmed the negative impact of hnRNP A1 knock-down on neuronal morphology by demonstrating decreased neurite outgrowth and branching, and increased toxicity. Additionally, we were able to show that hnRNP A1 knock-down led to the dysregulation of SG complex formation, confirmed through the reduction of SG number and size. These findings have implications for a wide array of neurologic diseases that involve hnRNP A1 mislocalization and emphasize the importance of proper hnRNP A1 functioning in neuronal cell health.

## Synthesis

Reviewing Editor: Deanna Smith, University of South Carolina Decisions are customarily a result of the Reviewing Editor and the peer reviewers coming together and discussing their recommendations until a consensus is reached. When revisions are invited, a fact-based synthesis statement explaining their decision and outlining what is needed to prepare a revision will be listed below. The following reviewer(s) agreed to reveal their identity: NONE.

Both reviewers felt that the work has new and interesting information that could shed light on mechanisms of hnRNA1 related diseases. However, there were some concerns which are listed for each reviewer after this synthesis. Please address each of their concerns. Please indicate whether impacts of knockdown prior to differentiation could complicate interpretations. Also please ensure that RNAseq data is publicly available. You should also address the statistical concerns of reviewer 2, and possibly consider using estimation statistics. A good site for analysis of data using estimation statistics can be found here: https://www.estimationstats.com

Reviewer 1 comments:

In “Knockdown of heterogeneous nuclear ribonucleoprotein A1 results in neurite damage, altered stress granule biology and cellular toxicity in differentiated neuronal cells” several interesting consequences resulting from hnRNPA1 kd are described. Intriguingly, neurite outgrowth and stress granules were impacted. The authors conducted sequencing to determine how hnRNPA1 targets were impacted and found that many of the DE transcripts physically associate with hnRNPA1. Overall, this is an interesting study and I have some minor comments. If these are clarified, my hope that this will improve the overall strength of the manuscript.

Given the core aim of this study is to examine the contribution of nuclear loss of function of hnRNPA1 (as opposed to cytosolic gain of function) to neuropathology, it may be useful to highlight how the findings of the present study compare to those investigating hnRNPA1 mislocalization and aggregation.

The methods for quantifying corrected total A1 nuclear fluorescence (Fig. 1D) and A1 cellular fluorescence (Fig 5C) are not described. Please include how the data were collected, quantified, and normalized.

The authors indicate that siRNA knockdown initiated 16 hrs prior to differentiation. Can the observations of the study be explained by alterations in cell identity (i.e. delay or failure to differentiate) following hnRNPA1 KD?

The fluorescence microscopy is concerning. Fig. 1C there is clear cytosolic fluorescence in the DAPI channel. The representative images for siNEG in 4A have very high background. The representative images on Fig. 5A show a high amount of blurring/haloing. This can cause artifacts in fluorescence measurements. With so much of this investigation hinging on fluorescence data, the authors should take care to properly optimize laser and acquisition settings to avoid artifacts.

The change in stress granule dynamics is interesting. Does hnRNPA1 depletion affect how neurons recover from stress or the transcripts that are shuttled to granules during arsenite treatment?

The methods appear to indicate that the stress granule experiments were performed on undifferentiated cells. This is inconsistent with the rest of the study.

Methods only list two fluorescent secondary antibodies, but three fluorescent tags were used in Fig. 5.

Is there a correlation between hnRNPA1 fluorescence (expression) and neurite size/branching? Could the authors include an analysis using the already collected images (similar to 5C)?

Fig. 5B the authors oversell the change in stress granule abundance as “substantially” greater in siNEG cells compared to siA1. The difference is 2 SGs

Fig. 5 Why the discrepancy in n for similar analyses presumably using the same images (n = 3 in B, D; n = 100 in C)?

Reviewer 2 comments:

In this study, authors modeled loss-of-function of an RNA binding protein hnRNP A1 by siRNA mediated knockdown in differentiated mouse Neuro-2a cells. Using unbiased RNA sequencing (RNA-seq) authors identified effect on steady state levels of a number of RNA, which was followed by bioinformatics and gene ontology (GO) analyses. This approach showed that hnRNP A1 is involved in important biological processes, such as RNA metabolism, neuronal function, neuronal morphology, neuronal viability, and stress granule (SG) formation. Authors then confirmed that hnRNP A1 knockdown results in reduction of neurite number and length, and found an increase in cell cytotoxicity and changes in SG formation. The authors’s experimental approach is adequate (e.g. appropriate markers were used) and results presented support main conclusions. I have only some minor suggestions:

1) Author may mention N2a cells in the title.

2) Authors may want to discuss in greater detail how knockdown of hnRNP A1 can mimic the hnRNP A1 mislocalization from the nucleus to the cytoplasm in neurodegenerative diseases.

3) Authors should be consistent in data presentation using graphs. All graphs should be presented as scatterplots.

4) Figure 1 legend is missing note what is the “B” panel representing.

5) Figure 3 statistics and significance should be re-confirmed. Possibly scatterplot representation will give more confidence in these results.

6) Are some of the RNAs found to be bound by hnRNP A1 more represented din GO terms shown in Figure 2?

7) 7) Given the toxicity, authors should discuss possibility that neurite differences may be secondary to possible lower cell density when hnRNP A1 is knocked down.

8) It is unclear from the manuscript if RNAseq data will be deposited as a source and publicly available.
